# Supplementary material for: Long-Term Safety of Prenatal and Neonatal Exposure to Paracetamol: A Systematic Review
Source: Int J Environ Res Public Health. 2022 Feb 14;19(4):2128. doi: 10.3390/ijerph19042128 (PMC8871754; doi:10.3390/ijerph19042128)
Supplement: Supplementary file 1 [file ijerph-19-02128-s001.zip › ijerph-1539865-supplementary.pdf]

Supplementary Table S1. Characteristics of Included Studies on the Long-Term Safety of Prenatal Paracetamol Exposure

Study	Design	Aim	Population	Sample Size	Exposure Assessment	Age* (years)	Exposure Time	BMI	Maternal Underlying Condition(s)	Maternal Comorbidity	Maternal SES	Dose (mg/kg), Dose Interval	Duration	Cumulative Dose (mg/kg)	Outcome (s) Assessed	Outcome Assessment Time	Method of Assessment	Results	Conclusions
Bauer 2013 [22]	Ecologic	Relationship of population weighted average of ASD prevalence rates and prenatal paracetamol use	20 English language studies examining health outcomes related to prenatal paracetamol use	N/A	Studies on prenatal paracetamol use	N/R	N/R	N/R	N/R	N/R	N/R		Paracetamol		ASD	N/R	Male participants of studies in the CDC's ASD Prevalence Summary	A country's average prenatal paracetamol use was correlated with its ASD prevalence (r = 0.80).	Biologic plausibility along with clinical evidence is linking paracetamol to ASD and abnormal NDV.
												N/R	N/R	N/R					
			N/A	N/A	N/A								Other Pharmacotherapy, Placebo or Standard Practice						
												N/R	N/R	N/R					
Stergiakouli 2016 [23]	Prospective Cohort	Association between prenatal paracetamol use and behavioural problems in offspring	Cases Mother-child pairs with prenatal paracetamol exposure	Exposed at ≤ 18 WGA: 4,415 Exposed at ≤32 WGA: 3,381	Self-reported, maternal questionnaire	29.1 (4.5)	≤18 WGA, ≤32 WGA	23.3 (3.9)	N/R	Psychiatric Illness: 300 (10.2)<br><br>Alcohol Use: 1,820 (59.9)<br>Smoking: 650 (21.3)	Low: 429 (16.3)<br><br>Middle: 1,121(42.6)<br><br>High 1,082(41.1)		Paracetamol		Behaviour problems	7 years	MSD Scale	Exposure at ≤18 WGA: ↑ conduct problems (RR: 1.20, 1.06-1.37) hyperactivity symptoms (RR: 1.23, 1.08-1.39).<br><br>Adjusted for prenatal and postnatal paracetamol use: ↑ hyperactivity symptoms (scores ≥7: RR: 1.18, 1.01-1.38)<br><br>Exposure at ≤32 WGA: ↑ emotional symptoms (RR: 1.29, 1.09-1.53) ↑ conduct problems (RR: 1.42, 1.25-1.62), hyperactivity (RR: 1.31, 1.16-1.49) ↑ total difficulties (RR: 1.46, 1.21-1.77).<br><br>Adjusted for prenatal and postnatal	Children with prenatal exposure to paracetamol have ↑ risk of behavior difficulties that are not explained by behavior or social factors linked to paracetamol use.
												N/R	N/R	N/R					
			Controls Mother-child pairs without prenatal paracetamol exposure	4,681		29.2 (4.5)	≤18 WGA ≤32 WGA	22.6 (3.5)		Psychiatric Illness: 283 (6.7)<br><br>Alcohol Use: 2,295 (53.5)<br><br>Smoking: 720 (16.7)	Low: 609 (15.8)<br><br>Middle: 1,631 (42.4)<br><br>High: 1,608 (41.8)		Other Pharmacotherapy, Placebo or Standard Practice						
												N/R	N/R	N/R					

																		paracetamol use: ↑ hyperactivity (scores ≥7: RR: 1.22, 1.04-1.43) ↑ total difficulties (scores ≥17, RR: 1.37, 1.07 - 1.75) ↑ conduct problems (scores ≥4, RR: 1.35, 1.13 - 1.6).	
Andersen 2012 [35]	Prospective Cohort	Risk of asthma with prenatal paracetamol exposure	Cases Mother-child pairs with prenatal paracetamol exposure	976	Prescription Database	N/R	30 days before the first day of LMP until delivery.	N/R	N/R	N/R	N/R		Paracetamol		Asthma	From birth until the date of asthma diagnosis, death, emigration or the end of cohort follow-up.	National Patient Registry	35 ↑ (aIRR: 1.35, 1.17-1.57) for children prenatally exposed to paracetamol as compared to unexposed children after adjustment. aIRR: 1.40 (1.16-1.71) for paracetamol exposure in the T1 and aIRR: 1.28 (1.02-1.62) for exposure in T2/T3.	There is a robust association between maternal prenatal paracetamol exposure and the risk of asthma in offspring.
												N/R	N/R	N/R					
			Controls Mothers-child pairs without prenatal paracetamol exposure	196, 084									Other Pharmacotherapy, Placebo or Standard Practice						
												N/R	N/R	N/R					
Bertoldi 2019 [24]	Prospective Cohort	Risk of NDV adverse outcomes with prenatal paracetamol exposure	Cases Cohorts of Viva and Pelotas studies: Mother-child pairs with prenatal exposure to paracetamol	Cases Viva T1/ T2: 837<br>T1 & T2: 487<br>Pelotas: T1/T2: 2,198<br>T1 & T2: 1,274	Maternal questionnaires and interviews; Electronic medical records	Viva: 32.5 (5.0)<br>Pelatos: 27.1 (6.6)	Viva: T1/ T2, T1 and T2<br>Pelotas: T1/ T2, T1 and T2<br>T1/T2/T3 T1, T2, and T3	Viva: 24.6 (5.1)<br>Pelatos: 25.8 (5.4)	N/R	N/R	N/R		Paracetamol		NDV outcomes	2-3 years	Performance based tests (PPVT and WRAVMA), observation assessments (INTER-NDA), and parental questionnaire	Viva: Exposure to Paracetamol in T1 and T2 associated with ↓ WRAVMA scores (β: −1.51, −2.92 - −0.10)<br>Pelatos: Exposure to paracetamol in T1 and T2 not associated with INTER-NDA motor scores (β: 0.02, −0.05-0.09) but	There is not strong evidence of a negative association between prenatal paracetamol use and cognition in early childhood.
												N/R	N/R	N/R					
			Control Cohorts of Viva and	Control Viva:		Viva: T1/ T2 32 (5.2)		Viva: T1 /T2	N/A	N/A	N/A		Other Pharmacotherapy, Placebo or Standard Practice						

			Pelotas studies: Mother-child pairs without prenatal exposure to paracetamol	T1/T2: 361<br><br>T1 & T2: 692<br><br>Pelotas T1/T2: 1,620<br><br>T1 & T2: 2,544<br><br>T1/T2/T3: 1,348<br><br>T1, T2 & T3: 3,044		T1 & T2 32 (5.1)<br><br>Pelotas: T1/T2 26.7 (6.7)<br><br>T1 & T2 26.9 (6.6)<br><br>T1/T2/T3 26.8 (6.6)<br><br>T1, T2 & T3 27 (6.6)		23.8 (4.4)<br><br>T1 & T2 24.5 (5.2)<br><br>Pelotas: T1/T2 25.4 (5.3)<br>T1 & T2 25.5 (5.3)<br><br>T1/T2/T3 25.4 (5.2)<br><br>T1,T2 & T3 25.6 (5.3)				N/R	N/R	N/R				associated with higher INTER-NDA total scores (β: 0.08, 0.01-0.16)	
Ji 2020 [26]	Prospective Cohort	Association between cord plasma biomarkers of prenatal paracetamol exposure and risk of ADHD and ASD	Cases Mother-child pairs with maternal cord paracetamol burden in the T1.<br><br>Mother-child pairs with maternal cord paracetamol burden in the T2.	Cases T1: 332<br><br>T2: 332	Maternal cord plasma	<20: 65 (11.85)<br><br>20-34: 728 (70.99)<br><br>≥ 35: 183 (17.15)	N/R	26.9 (6.5)	N/R	N/R	Annual Income: <$30,000: 475 (49.67)<br><br>>$30,000: 143 (14.35)<br><br>Unknown: 378 (37.95)		Paracetamol		ADHD, ASD, other DDs.	0-21 years	Electronic medical record	Second and third vs. first percentile of cord paracetamol burden associated with ↑ ADHD (OR for second 2.26: 1.40-3.69; OR for third 2.86: 1.77-4.67)<br><br>↑ASD (OR for second 2.14: 0.93-5.13; OR for third, 3.62: 1.62-8.60), which remained after adjustment for confounders.	Findings suggest prenatal paracetamol exposure is associated with an increased risk of ADHD and ASD.
												N/R	N/R	N/R					
			Controls Mother-child pairs with maternal cord paracetamol burden in the T3.	Controls T3: 332									Other Pharmacotherapy, Placebo or Standard Practice						
												N/R	N/R	N/R					
Garcia-Marcos, 2009 [36]	Epidemiologic Survey	Effect of prenatal paracetamol exposure on the prevalence of wheezing, modified by maternal asthma.s	Cases Pairs of mothers with asthma and their children exposed to prenatal paracetamol<br><br>(1) ≥ 1 in pregnancy<br><br>(2) ≥ 1/mo	Mothers with asthma and exposure: 34<br><br>Non-asthmatic mothers with exposure: 901	Self-reported, maternal questionnaire	N/R	N/R	N/R	N/R	N/R	N/R		Paracetamol		Wheezing	3-4 yo	Self-reported by maternal questionnaire	Significant association between prenatal paracetamol use ≥ 1 and childhood wheezing, both unadjusted (OR 1.94: 1.34-2.79) and adjusted (OR 1.74: 1.15-2.61).	The frequent usage of paracetamol during pregnancy is associated with the prevalence of wheezing in offspring during preschool years. Asthma in
												N/R	≥ 1<br><br>≥ 1/m	N/R					

			pregnancy																the mother might modify this association.
			Controls<br>Pairs of mothers with asthma and their children never exposed to prenatal paracetamol	Asthmatic mothers without exposure:<br>31<br><br>Non-asthmatic mothers without exposure:<br>775									Other Pharmacotherapy,<br>Placebo or Standard Practice						
												N/R	N/R	N/R					
Golding<br>2020<br>[27]	Prospective Cohort	Association between prenatal paracetamol exposure and childhood behavioural and cognitive outcomes.	Cases<br>Mother-child pairs with prenatal paracetamol exposure	Cases<br>5,279	Self-reported, maternal questionnaire	N/R	Total Cohort:<br>18-32 WGA	23.3<br>(SD, N/R)	N/R	Asthma:<br>1,295 (13.6)<br><br>Back Pain:<br>5,008 (52.6)<br><br>Migraine:<br>5,113 (53.7)	N/R		Paracetamol		Cognitive and behaviour outcomes	0.5-17 years	Cognition<br>IQ test at 8 and 15 years<br><br>Temperament maternal questionnaire, Carey scales and EAS[7] scale<br><br>Behavioural maternal and teacher questionnaire	↓ adaptability at 6 months (AMD 0.33: 0.05-0.60)<br><br>↓ persistence at 24 months (AMD 0.36: 0.12-0.60)<br><br>↑ hyperactive behaviour scores at 42 months (AMD 0.16: 0.07-0.25) and 47 months (AMD 0.22: 0.10-0.33).<br><br>↓ attention in school for 7-8 years<br>(MD 0.45: 0.11-0.79)<br><br>Conduct problems at 42 months (AMD 0.22: 0.10-0.33) and 47 months (AMD 0.08: 0.01-0.15)<br><br>Behaviour difficulties at 42 months (AMD 0.54: 0.26-0.82) and 47 months (AMD 0.31: 0.08-0.53)	Prenatal paracetamol exposure is associated with child attention and hyperactivity until 7 years, and conduct problems in boys until 9 years.
												N/R	N/R	N/R					
			Controls<br>Mother-child pairs without prenatal paracetamol exposure	Controls<br>6, 746				22.6<br>(SD, N/R)		Asthma:<br>942 (9.9)<br><br>Back Pain:<br>4,065 (42.7)<br><br>Migraine:<br>3,475 (36.5)			Other Pharmacotherapy,<br>Placebo or Standard Practice						
												N/R	N/R	N/R					
Ji<br>2018<br>[25]	Prospective Cohort	Association between maternal plasma biomarkers of paracetamol	Cases<br>Mother-child pairs with maternal paracetamol	Above median<br>668<br><br>Below median<br>630	Maternal blood samples	< 35 :<br>965<br><br>≥ 35:<br>215	N/R	< 18:<br>418<br><br>19-24:<br>514<br><br>25-30:	N/R	Smoking:<br>112 (9.6)<br><br>Alcohol Use:<br>84 (7.1)	Maternal Education<br>< college:<br>1,033 (87.6)<br><br>≥ college degree:		Paracetamol		ADHD, ASD, other DDs.	Median age<br>7 years	Electronic medical record	Below median and above median levels of maternal paracetamol burden → 58 and 88 ↑ odds	Maternal plasma biomarkers of paracetamol are associated with ↑ risk
												N/R	N/R	N/R					

		and ADHD in offspring.	biomarkers of<br>(1) above median<br>(2) below median					337<br>>30: 288			145 (12.4)		Other Pharmacotherapy, Placebo or Standard Practice					of ADHD diagnosis, (OR 1.58: 1.02-2.46 and OR 1.88: 1.18-3.00).	of ADHD diagnosis
			Controls<br>Mother-child pairs with maternal paracetamol biomarkers of "no detection"	Controls 1,062								N/R	N/R	N/R					
Bakkeheim 2011 [44]	Prospective Cohort	Association between prenatal paracetamol exposure and allergic disease in school aged children	Cases<br>Mother-child pairs with prenatal paracetamol exposure	T1: 31<br>T2/T3: 32	Self-reported, maternal questionnaire	N/R	T1 and T2	N/R	Maternal pain or fever	N/R	N/R	N/R	Paracetamol<br>N/R	N/R	Primary outcomes: current asthma, allergic sensitization, allergic rhinitis<br>Secondary outcomes: history of asthma, current wheeze, FeNO $\geq$ 16.7 (ppb), mild to moderate bronchial hyperresponsiveness, severe bronchial hyperresponsiveness	10	Parental interview with central ISAAC questions<br>Lung function testing, exercise tests, skin prick tests and serum analysis.	T1:<br>↑ Current allergic rhinitis (OR 2.30: 1.96-4.97).<br>T2/T3<br>↑ high FeNO levels in 10 year olds (OR 0.64: 1.31, 11.37).	Paracetamol exposure in pregnancy was associated with allergic rhinitis, but not with asthma or allergic sensitization at 10 years.
			Controls<br>Mother-child pairs without prenatal paracetamol exposure	972								N/R	Other Pharmacotherapy, Placebo or Standard Practice<br>N/R	N/R					
Piler 2018 [37]	Prospective Cohort	Association between prenatal paracetamol exposure and asthma in offspring.	Cases<br>Mother-child pairs with prenatal exposure to paracetamol	170	Self-reported, maternal questionnaire	Cases:<br><19: 0.8%<br>20-24: 1.2%<br>25-30: 1.5%<br>>31: 2.2%	<20 WGA	<18.5: 1.5%<br>18.5-24.9: 1.3%<br>25-29.9: 1.4%<br>>30: 2.2%	N/R	N/R	Secondary Education: 6.8%<br>Married: 6.1%	N/R	Paracetamol<br>N/R	N/R	Asthma	3, 5, 7 and 11 years	Health records and ICD codes	No statistically significant relationship in prenatal paracetamol exposure alone (aOR 1.12: 0.25, 4.98).	The combination of prenatal and postnatal paracetamol exposure leads to higher risk of asthma development.
													Other Pharmacotherapy, Placebo or Standard Practice						
			Controls<br>Mother-child pairs	Without paracet		Controls:<br><18.5		<18.5: 29.6%			Secondary								

			(1) without prenatal paracetamol exposure<br><br>(2) with prenatal exposure to paracetamol and aspirin<br><br>(3) with prenatal exposure to aspirin	amol exposure: 3,324<br><br>With paracetamol and aspirin exposure: 97<br><br>With aspirin exposure: 532		18.5-24.9:<19: 41.6%<br>20-24: 35.1%<br>25-30: 25.0%<br>>31: 30.9%		18.5-24.9: 31.0%<br>25-29: 32.9%<br>>30: 34.8%			Education: 29.3%<br><br>Married: 31.6%	Aspirin<br>N/R	Aspirin<br>N/R	Aspirin<br>N/R					
Shaheen 2002 [49]	Longitudinal Cohort	Association between prenatal paracetamol use and risk of wheezing and eczema	Cases Mother-child pairs with prenatal paracetamol exposure	9,400	Self-reported, maternal questionnaire	N/R	<20 WGA<br><br>20-32 WGA	N/R	N/R	N/R	N/R		Paracetamol		Wheezing, eczema	Wheezing: 43 months<br><br>Eczema: 30 months	Maternal questionnaires at 6 months and every 12 months thereafter.	Late and moderate use: ↑ in risk of 12-month prevalence of wheezing (OR 1.12: 0.98-1.28) ↑ risk of persistent wheezing (aOR 2.34: 1.24-4.40, p = 0.008).<br><br>Frequent use (adjusted OR 2.10: 1.30-3.42, p = 0.003).	Frequent use of paracetamol in late pregnancy may increase the risk of wheezing in the offspring.
												N/R	N/R	N/R					
			Controls Mother-child pairs (1) without prenatal paracetamol exposure<br><br>(2) with prenatal aspirin exposure										Other Pharmacotherapy, Placebo or Standard Practice						
												N/R	N/R	N/R					
Ernst 2019 [45]	Longitudinal Cohort	Association between prenatal paracetamol exposure and timing of pubertal development	Cases Mother-child pairs with prenatal paracetamol exposure	8,606	Maternal enrollment form and computer-assisted telephone interviews	Cases: 30.6 (4.4)<br><br>Controls 30.7 (4.4)	<12 WGA,<br><br>13-24 WGA,<br><br>>25 WGA	23.4 (4.4)	N/R	Fever: 2,833 (33)<br><br>Muscle/Joint Disease: 1,565 (18.2)<br><br>Inflammation/ Infection: 1,483 (17.4)	Low SES: 2,821 (32.8)<br><br>Alcohol Use T1: ,4288 (49.9)<br><br>Number cigarettes/ day smoked T1: 2.2 (4.5)		Paracetamol		Pubertal development	11.5 years and every 6 months until full sexual maturation or 18 years	Self-reported using the British Avon Longitudinal Study of Parents and Children questionnaire.	Girls: earlier age in months of pubertal development, (AMD −1.2: −2.2-−0.2), and acne (AMD −1.7, −2.7-−0.6).<br>.<br>Pubertal hair (AMD −1.7: −3.2-−0.1), axillary hair growth (AMD −3.0: −5.1-−0.9), and acne (AMD −2.8: −5.0-−0.5).	Prenatal paracetamol use may have long-term effects on female offspring pubertal development.
												N/R	N/R	N/R					
			Controls Mother-child pairs without prenatal paracetamol exposure	7,216				24.2 (4.7)		Fever: 1,551 (21.5)<br><br>Muscle/ Joint Disease: 965 (13.4)<br><br>Inflammation/ Infection: 825 (11.7)	Low SES: 2,375 (33.0)<br><br>Alcohol Use T1: 3,349 (46.7)<br><br>Number cigarettes/ Day T1: 1.8 (4.2)		Other Pharmacotherapy, Placebo or Standard Practice						
												N/R	N/R	N/R					
			Cases	Cases			T1, T2, T3	N/R	Pain	N/R	N/R		Paracetamol		Eczema		Maternal		

Jedrychowski 2011 [39]	Prospective Cohort	Association between low prenatal exposure to paracetamol and eczema in early childhood and interaction with prenatal exposure to pollutants	Mother-child pairs with prenatal paracetamol exposure in T1 or T2 or T3	T1: 40 T2: 40 T3: 73	Self-reported by maternal interview	28.82 (3.39)			256 (79.8) Fever 67 (20.8).			N/R	N/R	T1: 200 (500-1500) T2: 1600 (500-1100) T3: 2,200 (500-8000) T1/T2/T3: 2,600 (500-1600)		3-24 months: every 3 months 24-60 months: every 6 months	interviews	Prenatal exposure to paracetamol ↑ the risk of eczema by 20. Prenatal exposure to particulate matter ↑ eczema risk by 6. Joint exposure to paracetamol and higher prenatal particulate matter was significant and doubled the risk of eczema symptoms (HR 2.07: 1.01-4.34).	Very small doses of paracetamol in pregnancy may affect the occurrence of eczema in early childhood, but only with co-exposure to high concentrations of fine particulate matter.
													Other Pharmacotherapy, Placebo or Standard Practice						
			Controls: Mother-child pairs without prenatal paracetamol exposure in T1 or T2 or T3	Controls T1: 283 T2: 283 T3: 248								N/R	N/R	N/R					
Vlenterie 2016 [28]	Propensity Score Matched Cohort	Association between NDV impairment with prenatal exposure to paracetamol	Cases Mother-child pairs with paracetamol exposure	20,749	Self-reported, maternal questionnaire	<25: 155 25-29: 594 30-34: 805 ≥ 35: 345	N/R	<18: 53 18-25: 1,023 ≥25: 789	N/R	Depression 298 (1.43) Smoking 302 (1.45) Alcohol Use 437 (2.10)	Married or cohabiting: 1,826 (5.93) 13-17 years of education: 777 (3.74)		Paracetamol		Psychomotor or behaviour/ temperament problems	18 months	The Child Behaviour Checklist: Ages and Stages Questionnaire Temperament problems: short-form of EAS.	Long-term prenatal paracetamol exposure associated with delayed age of starting to walk (OR 1.35: 1.07-1.70) and communication problems (OR 1.38: 0.98-1.95) Weak association with gross motor impairment (OR 1.28: 0.83-1.98), sociability problems (OR 1.1: 0.92-1.51) and shyness (OR 1.20: 0.86-1.66)	Long-term prenatal exposure to paracetamol associated with increased risk of motor milestone delay and impaired communication at 18 months.
												N/R	N/R	N/R					
			Controls Mother-child pairs without paracetamol exposur	30,451		<25: 3,069 25-29: 10,183 20-34: 11,870 >35: 5,329		<18: 939 18-25: 20,067 ≥25: 8,618		Depression 2,441 (8) Smoking 26,162 (85.91) Alcohol Use: 4,901 (16.09)	Married or cohabiting: 392 (1.28) 13-17 years of education: 12,217 (40.12)		Other Pharmacotherapy, Placebo or Standard Practice						
												N/R	N/R	N/R					
Fisher 2016 [46]	Prospective Cohort	Relationship between prenatal paracetamol intake and male infant genital development	Cases Mother-male child pairs with prenatal paracetamol exposure	225	Self-reported, maternal questionnaire	33.5	<8 WGA 8-14 WGA >14 WGA	23.5 [21.5, 26.0]	Headache 133 (59) Infection 41(18.3)	Smoking: 8	Caucasian: 129 Index of multiple deprivation 7.7 [6.4, 10.5]		Paracetamol		Male: AGD, penile length, testicular descent distance, cryptorchidism Female: AGD	0, 3, 12, 18, and 24 months.	Physical examination	Exposure at 8–14 WGA associated with shorter AGD (SD: 0.27, 0.06–0.48).	Prenatal paracetamol exposure during 8–14 weeks of gestation associated with shorter AGD to 24 months.
												N/R	N/R	3.0 (0.5-360.0).					
			Controls Mother-male child pairs without prenatal paracetamol exposure	456		33.5		22.8 [20.9, 26.0]	Headache N/R Infection: N/R	Smoking: 14	Caucasian: 297 Index of multiple deprivation 7.6 [6.4, 11.6]		Alternative Pharmacotherapy						
												N/A	N/A	N/A					

Lind 2017 [47]	Prospective Cohort	Association between prenatal analgesic exposure and anogenital distance in offspring	Cases Mother-child pairs with prenatal paracetamol exposure	Paracetamol exposure only: 365	Self-reported, maternal questionnaire	30.9	T1/T2	<18.5: 29 (2.8)<br><br>18.5-24.9: 638 (62.1)<br><br>25-29.9: 236 (23.0)<br><br>>30: 124 (12.1)	Headache: 498 (49.4)<br><br>Migraine: 48 (4.8)<br><br>Influenza: 67 (6.7)<br><br>Cold: 415 (41.1)<br><br>Muscle-Joint Pain: 223 (22.2)	Smoking: 11 (3)	Tertiary Education: 702 (69.2)<br><br>Western Ethnicity: 977 (95.3)	Paracetamol			AGD	3 months	Physical exam	Prenatal NSAID and paracetamol exposure associated with shorter AGD in male offspring (AGD: 4.1 mm, 6.4-1.7)	The negative association between prenatal analgesic exposure (NSAIDs only, and NSAIDs and paracetamol, but not paracetamol alone) and AGD in male offspring suggests disruption of androgen action.
												N/R	N/R	N/R					
			Controls Mother-child pairs<br><br>(1) With prenatal analgesic exposure<br><br>(2) With prenatal paracetamol and other analgesic exposure<br><br>(3) With prenatal exposure to other analgesics	No analgesic exposure: 617<br><br>Paracetamol and other analgesic exposure: 34<br><br>Other analgesics only: 11						Smoking: 14 (2)		Other Pharmacotherapy, Placebo or Standard Practice							
												N/A	N/A	N/A					
Persky 2008 [40]	Cohort**	Association between prenatal paracetamol exposure and wheezing and allergic symptoms in first year of life	Cases Mother-child pairs with prenatal exposure to paracetamol<br><br>(1) early pregnancy<br><br>(2) middle to late pregnancy	Early pregnancy: 344<br><br>Middle to late pregnancy: 342	Self-reported, maternal questionnaire	25.8 [15-43]	T1: 16-27 WGA<br><br>T2: 28-36 WGA	N/R	N/R	N/R	Mexican Ethnicity: 225 (65.2)<br><br>Language spoken, Spanish: 175 (50.7)<br><br>Less than high school education: 145 (42.3)<br><br>Worked outside the home: 102 (29.6<br><br>Married: 163 (47.3)	Paracetamol			Wheezing, wheezing/ coughing that disturbed sleep, emergency department visits, hospitalization, asthma	1, 3, 6, 9 and 12 months.	Parental self-report at study visits and study telephone calls	Paracetamol use in middle to late but not early pregnancy was significantly related to wheezing (OR: 1.8, 1.1-3.0) and to wheezing that disturbed sleep (OR 2.1; 1.1-3.8) in the first year of life after controlling for confounders..	Paracetamol use in middle to late but not early pregnancy may be related to respiratory symptoms in the first year of life.
												N/R	N/R	N/R					
			Controls Mother-child pairs with<br><br>(1) prenatal exposure to aspirin<br><br>(2) prenatal exposure to ibuprofen	Aspirin exposure: 9<br><br>Ibuprofen exposure: 11								Other Pharmacotherapy, Placebo or Standard Practice							
												N/R	N/R	N/R					
Petersen 2018 [34]	Prospective Cohort	Association between prenatal exposure to paracetamol,	Cases Mother-child pairs with prenatal	91,015	Self-reported, maternal questionnaire	Total Cohort ≤24: 19,318	First trimester<br><br>Second trimester	<18.5: 6,702 (3.6)	RTI: 78,412 (42.2)<br><br>Fever:	Rheumatoid arthritis	Total Cohort: Employed: 144,997 (78.1)	Paracetamol			Overall CP, unilateral spastic CP	1 to 6 years	Paediatrician Diagnosis	↑ risk of overall CP (aOR: 1.3, 1.0-1.7)	Children with T2 paracetamol exposure have ↑ risk
												N/R	N/R	N/R					

		aspirin or ibuprofen and risk of CP in offspring	paracetamol exposure<br><br>Controls Mother-child pairs with prenatal aspirin exposure<br><br>Mother-child pairs with prenatal ibuprofen exposure	Aspirin: 5,746<br><br>ibuprofen: 7,358		25-29: 66,037<br><br>30-34: 70,268<br><br>≥35: 29,994	Third trimester	18.5-24.9: 120,415 (64.9)<br><br>≥ 25: 54,341 (29.3)	32,078 (17.3)<br>UTI<br>21,158 (11.4)		Not Employed: 13,785 (7.4)<br><br>Student: 22,083 (11.9)<br><br>Smoking: 22,444 (12.6)	Other Pharmacotherapy, Placebo or Standard Practice<br>N/R	N/R	N/R				↑ risk of unilateral spastic CP (aOR: 1.5, 1.0-2.2).<br><br>↑ risk of unilateral spastic CP with T1 exposure (aOR: 1.6, 1.0-2.5).	of unilateral spastic CP.
Rebordosa 2018 [41]	Prospective Cohort	Association between prenatal paracetamol exposure and asthma or wheezing in early childhood	Cases Mother-child pairs with prenatal paracetamol exposure	Cases 18 month cohort: 54,530<br><br>7 year cohort: 9,546	Self-reported, maternal questionnaire and interviews	T1/T2/T3	18 month cohort: <24: 5,525<br><br>25-29: 25,601<br><br>30-35: 25,076<br><br>≥36: 10,147<br><br>7 year cohort: <24: 1,059<br><br>25-29: 4,732<br><br>30-35: 5,041<br><br>>36: 1,864	N/A	Pain: 44,139 {91}<br><br>Fever: 15,897 {32}<br><br>Infection/ Inflammation 7,900 {16}<br><br>Muscle/Joint Disease: 9,591 {20}	Asthma: 8m507 (9.4)<br><br>Fever 24,861 (27.5)<br><br>Muscle/Joint Disease: 15,169 (16.8)<br><br>Inflammation/ Infection: 12,439 (14.1)<br><br>Hypertension 10,189 (11.6)<br><br>Allergy: 27,546 (30.9)<br><br>Eczema: 7,108 (12.3)	High SES: 57,734 (66.8)<br><br>Medium SES: 25,236 (29.2)<br><br>Low SES: 3480 (4.0)	Paracetamol<br>N/R	N/R	N/R	18 months: asthma/ bronchitis; wheezing ever; hospitalization for asthma<br><br>7 years: asthma, wheezing in last 12 months; transient wheezing; persistent wheezing; late onset wheezing.	18 months or 7 years of age	18 months Maternal self-report<br><br>7 years Maternal interview, International Study of Asthma and Allergies in Childhood questionnaire, Danish National Hospital Registry	18 month ↑ wheezing and asthma (RR: 1.11, 1.08-1.14)<br><br>7 years ↑ wheezing in last 12 months (RR: 1.29, 1.11-1.51)<br><br>↑ persistent wheezing (RR: 1.45, 1.13-1.85)<br><br>8 months Adjusted ↑ asthma or bronchitis (RR: 1.20, 1.15–1.24).<br><br>↑ hospitalization for asthma (HR: 1.24, 1.11–1.38<br><br>↑ asthma or bronchitis (HR: 1.22, 1.10–1.36).<br><br>7 years ↑ asthma (RR: 1.22 , 1.11–1.34).<br><br>↑ risk for wheezing affecting sleep (RR: 1.27, 1.03–1.57)	Prenatal paracetamol exposure has moderate associations with asthma
			Controls Mother-child pairs without prenatal paracetamol exposure	Controls 18 month cohort: 30,129<br><br>7 year cohort: 5,981								Other Pharmacotherapy, Placebo or Standard Practice<br>N/R	N/R	N/R					
Ystrom, 2017 [29]	Prospective Cohort	Association between prenatal paracetamol exposure and	Cases Mother-child pairs with prenatal paracetam	Cases: 52,707	Self-reported, maternal questionnaire	N/R	6 months before pregnancy	N/R	Fever/ Infections and pain. 23 {51}	ADHD, anxiety, depression	N/R	Paracetamol<br>N/R	N/R	N/R	ADHD	3 years until diagnosis	Norwegian Patient Registry	Paracetamol Modest association between any prenatal maternal	Long-term prenatal paracetamol exposure is associated
												Other Pharmacotherapy,							

		ADHD in offspring	ol exposure				0-4, 5-8, 9-12, 13-16, 17-20, 21-24, 25-28, > 29, >30 WGA					Placebo or Standard Practice						paracetamol use in T1 (HR: 1.07, 0.96-1.19), T2 (HR: 1.22, 1.07-1.38) and T3 (HR: 1.27, 0.99-1.63).	with ADHD, even after confounder adjustment.
			Controls Mother-child pairs without prenatal paracetamol Exposure	Controls: 60,266								N/R	N/R	N/R					
Liew, 2016 [30]	Prospective Cohort	Association between prenatal paracetamol exposure and behaviour problems and HKDs in offspring.	Cases Mother-child pairs with prenatal paracetamol exposure	Total Cohort: 1,491	Self-reported, maternal interview	30.8 (4.4)	Trimesters 1, 2 and 3	<25: 629 (71.3)<br><br>25-30: 164 (18.6)<br><br>≥30: 69 (7.8)	Fever: 329 (37.2)<br><br>Pain: 105 (11.9)<br><br>Infection/ Inflammation: 115 (13.1)	Psychiatric Illness: 107 (12.2)	Years of Education: 13.1 (1.9)	Paracetamol			Behaviour problems, HKDs	ADHD Behaviors: 7 years<br><br>HKDs: 5 years<br><br>ADHD Medication Use: Timing of Assessment N/R	Parental completion of SDQ, Danish National Hospital Registry, Danish Psychiatric Central Registry, Danish Prescription Registry	Prenatal paracetamol exposure associated with risk of HKD (HR: 1.37, 1.19-1.59), use of ADHD medications (HR: 1.29, 1.15-1.44), or ADHD-like behaviors at 7 years (Rr: 1.13, 1.01-1.27)	Prenatal paracetamol exposure appears to be associated with increased risk of behavioural problems and HKDs.
												N/R	N/R	N/R					
			Controls Mother-child pairs without prenatal paracetamol exposure			30.8 (4.2)		<25: 449 (73.6)<br><br>25-30: 106 (17.4)<br><br>≥30: 44 (7.2)	Fever: 141 (23.1)<br><br>Pain: 48 (7.9)<br><br>Infection/ Inflammation: 47 (7.7)	Psychiatric Illness: 49 (8.0)	Years of Education: 13.3 (1.9)	Other Pharmacotherapy, Placebo or Standard Practice							
												N/R	N/R	N/R					
Rifas-Shiman, 2020 [31]	Prospective Cohort	Association between prenatal paracetamol and ibuprofen exposure and executive function and behaviour problems in offspring	Cases Mother-child pairs with prenatal paracetamol exposure	Total Cohort: 1,225	Self-reported, maternal interview	32.2 (5.2)	Early pregnancy (median 9.9 WGA),<br><br>mid pregnancy (median 27.9 WGA)	N/R	N/R	Depression: 116 (9.5)	College degree or higher: 845 (68.9)<br><br>Income > $70,000: 735 (60.1)<br><br>Caucasian Ethnicity: 790 (64.5)<br><br>Smoking 118 (9.7)	Paracetamol			Executive function and behaviour problems	First year of life, mid childhood (median 8 years)	BRIEF and SDQ	↑ (≥10 versus <10 times) prenatal paracetamol exposure associated with ↑ parent - rated BRIEF scores (β: 1.64 points, 0.59, 2.68).	Prenatal exposure to paracetamol is associated with poorer executive function in children.
												N/R	N/R	N/R					
			Controls Mother-child pairs (1) without prenatal paracetamol exposure<br><br>(2) with prenatal ibuprofen exposure									Other Pharmacotherapy, Placebo or Standard Practice							
												N/R	N/R	N/R					
Streissguth,	Prospective Cohort	Association between	Cases	Total Cohort:		N/R	22 WGA	N/R	Headache: 550 (36)	N/R	N/R	Paracetamol			N/R	4 years	WPPSI;	Paracetamol was not	Prenatal paracetamo

1987 [32]		prenatal paracetamol/ aspirin exposure and IQ/attention in offspring	Mother-child pairs with prenatal paracetamol exposure	1,529<br><br>Follow-up at 4 years: 421	Self-reported, maternal interview				Other Pain: 45 (3)<br><br>Infection: 15 (1)			N/R	N/R	N/R			Vigilance paradigm assessment in a laboratory	significantly related to child IQ or attention (MD in IQ among exposed vs. unexposed children was 0.28, p = 0.61).	l exposure was not associated with child IQ or attention at 4 years.
												Other Pharmacotherapy, Placebo or Standard Practice							
			Controls Mother-child pairs (1) without prenatal paracetamol exposure<br><br>(2) with prenatal aspirin exposure									N/R	N/R	N/R					
Thompson 2014 [33]	Prospective Cohort	Association between prenatal paracetamol use and ADHD in offspring	Cases Mother-child pairs with prenatal paracetamol exposure	Cases (437) 49.8	Self-reported, maternal questionnaire	N/R	N/R	N/R	N/R	N/R	N/R	Paracetamol			ADHD symptoms	7 and 11 years	Self-report by maternal and child questionnaire (SDQ, CPRSR) and interview	↑ total difficulty scores (MD: 1.2, 0.4, 2.0 and MD: 0.8, 0.0-1.6).<br><br>↑ risk of ADHD, hyperactive impulsive scores (MD: 2.0, 0.5-3.5)	Prenatal paracetamol exposure increases risk of ADHD-like behaviours.
												N/R	N/R	N/R					
												Other Pharmacotherapy, Placebo or Standard Practice							
			Controls Mother-child pairs<br><br>(1) without prenatal paracetamol exposure<br><br>(2) with prenatal exposure to other drugs	Anti-inflammatory drugs: 11 (1.3)<br><br>Aspirin: 46 (5.3)<br><br>Antacid: 151 (17.4)<br><br>Antibiotics: 204 (23.5)								N/R	N/R	N/R					
Magnus 2016 [42]	Prospective Cohort	Association between prenatal paracetamol exposure and asthma in offspring	Cases Mother-child pairs with prenatal paracetamol exposure	Assessed at<br><br>3 years: 53,169<br><br>7 years: 25,394	Self-reported, maternal questionnaire	<25: 1,432 (30.1)<br><br>25-29: 4,927 (27.8)<br><br>30-34: 5,876 (27.8)<br><br>≥35: 2,606 (27.3)	≤18 WGA<br><br>≤30 GA<br><br>< 6 months postpartum	<18.5: 387 (25.5)<br><br>18.5-24.9: 9,280 (26.8)<br><br>25-29.9: 3,385 (30.1)<br><br>≥30: 1,485 (32.1)	Pain, RTI /influenza, fever	Asthma: 1,222 (31.9)	High school education 4,238 (29.9)<br><br>Smoking: 1,260 (31.8)	Paracetamol			Asthma	3 & 7 years	Self-reported maternal questionnaire; Prescription registry	3 years ↑ asthma (aRR: 1.13, 1.02-1.25)<br><br>7 years ↑ asthma (aRR: 1.27 1.09–1.47) ↑ dispensed asthma medication (aRR: 1.17, 1.04–1.31)	Prenatal paracetamol exposure independently associated with asthma-related outcomes at 3 and 7 years.
												N/R	N/R	N/R					
			Controls Mother-child pairs without prenatal			<25: 1,712 (36.0)		<18.5: 633 (41.7)		Asthma: 1,091 (28.5)	High school education 5,330 (37.6)	Other Pharmacotherapy, Placebo or Standard Practice							
												N/R	N/R	N/R					

			paracetamol exposure			25-29: 6,522 (36.8)<br><br>30-34: 7,820 (37.0)<br><br>≥35: 3,857 (40.4)		18.5-24.9: 13,573 (39.2)<br><br>25-29.9: 3,800 (33.8)<br><br>≥30: 1,420 (30.7)			Smoking: 1,295 (32.6)								
Sordillo 2015 [43]	Prospective Cohort	Association between prenatal antipyretic exposure and asthma-related outcomes in offspring	Cases Mother-child pairs with prenatal paracetamol exposure	992	Self-reported, maternal questionnaire	32.2 (5.2)	Pregnancy	25.8 (4.3)	N/A	Asthma: 195 (13.1)	1,036 (69.7) Caucasian<br><br>853 (62.7) household income >$70,000	Paracetamol			Wheeze, asthma, allergen sensitization	3-5 yo, 7-10 yo	Wheeze & asthma: self-report maternal questionnaire<br><br>Allergen sensitization: serum IgE	Prenatal paracetamol exposure associated with increased asthma in early but not mid-childhood (OR: 1.26, 1.02-1.58), However association was reduced after adjusting for confounder (OR: 1.21, 1.00-1.48)	Adjustment for respiratory infections in early life substantially diminished associations between infant antipyretics and early childhood asthma. Respiratory infections should be accounted for in studies of antipyretics and asthma, to mitigate bias due to confounding by indication.
												N/A	N/A	N/A					
			Controls Mother-child pairs<br><br>(1) without prenatal paracetamol exposure<br><br>(2) with prenatal ibuprofen exposure	Without prenatal paracetamol exposure: 430<br><br>With prenatal ibuprofen exposure: 247								Alternative Pharmacotherapy							
												N/A	N/A	N/A					
Snijder, 2011 [48]	Prospective Cohort	Association between prenatal analgesic exposure and cryptorchidism/hypospadias	Cases Mother-child pairs with prenatal paracetamol exposure	2388 (75)	Self-reported, maternal questionnaire	29.96 (5.24)	Periconception;<br><br><14 WGA;<br><br>14-22 WGA;<br><br>20-32 WGA	24.56 [4.30]	N/R		Mid education level: 1,530 (48.4)<br><br>From Netherlands: 1,667 (52.3)<br><br>Smoking: 458 (14.4)<br><br>Alcohol Use: 1170 (36.7)	Paracetamol			Cryptorchidism, hypospadias	30 months	Physical exam	Use in T2 ↑ risk of cryptorchidism primarily due to paracetamol (aOR: 1.89, 1.01-3.51)	Prenatal exposure to paracetamol increases risk of cryptorchidism in offspring
												N/R	N/R	N/R					
			Controls Mother-child pairs<br><br>(1) without prenatal analgesic exposure<br><br>(2) with prenatal NSAID exposure<br><br>(3) with prenatal exposure	NSAID: 414 (13)<br><br>Other analgesic: 382 (12)								Other Pharmacotherapy, Placebo or Standard Practice							
												N/R	N/R	N/R					

			to other analgesics																
Shaheen 2010 [36]	Longitudinal Cohort	To explore potential interactions between prenatal and infant acetaminophen exposure and antioxidant genotypes on childhood asthma	Cases Mother-child pairs with prenatal paracetamol exposure<br><br>Controls Mother-child pairs without prenatal paracetamol exposure	Total Cohort 13,988	Self-reported, maternal questionnaire	27.2 (5)	<18-20 WGA<br><br>20-32 WGA	23.2 [4.1]	N/R	N/R	Car in household: 10,072 (90.8)<br><br>Non-White mother: 243 (2.2)<br><br>Married:: 8,805 (79.4)	Paracetamol<br>N/R<br><br>Other Pharmacotherapy, Placebo or Standard Practice<br>N/R	N/R<br><br>N/R	N/R<br><br>N/R	Wheezing, asthma, eczema, hayfever.<br><br>Lung function<br><br>Atopy (positive reaction to D. pteronyssinus, cat or grass)	7, 7.5 and 8.5 years	Maternal questionnaire, spirometry, serum IgE.	Risk of asthma and wheezing associated with early gestation acetaminophen exposure was increased when maternal copies of the minor T allele of Nrf2 were present (P interaction 0.02 and 0.04, respectively). Risk of asthma associated with late gestation exposure was higher when maternal GSTT1 genotype was present, rather than absent (P interaction 0.006), and risk of wheezing was increased when maternal GSTM1 was present (P interaction 0.04). Presence of GSTM1 in mother and child both increased risk of asthma and wheezing associated with late gestation acetaminophen Exposure	Maternal antioxidant gene polymorphisms may modify the relation between prenatal acetaminophen exposure and childhood asthma, strengthening evidence for a causal association.

*Maternal age; **Treated as an observational cohort study

Note: Mean (SD), Median [IQR], N {Percent}; (Effect Estimate: 95%CI-95% CI) or Effect Estimate: (95% CI-95% CI); ↑, increased; ↓, decreased; <, less than; >, greater than; ≤, less than or equal to; ≥, greater than or equal to

ADHD, attention deficit hyperactivity disorder; AGD, anogenital distance; aIRR, adjusted incidence rate ratio; AMD, adjusted mean difference; aOR, adjusted odds ratio; aRR, adjusted relative risk; ASD, autism spectrum disorder; BREIF, behavior rating inventory of executive function; CPRSR, conners' parent rating scale-revised; CI, confidence interval; CP, cerebral palsy; DD, developmental disorder; EAS, Emotionality, Activity, and Sociability; FeNO, fractional exhaled nitric oxide; HKD, hyperkinetic disorder; HR, hazard ratio; ICD, international classification of diseases; INTER-NDA, INTERGROWTH-21stneurodevelopment assessment; ISAAC, international study of asthma and allergies in childhood; IQ, intelligence quotient; IQR, interquartile range; LMP, last menstrual period; MD, mean difference; MSD, motor social development; N/A, not applicable; NDV, neurodevelopment; NSAID, non-steroidal anti-inflammatory drugs; PPVT, peabody picture vocabulary test; N/R, not reported; OR, odds ratio; RR, relative risk; Rr, risk ratio; RTI, respiratory tract infection; SD, standard deviation; SDQ, strength and difficulties questionnaire; SES, socioeconomic status; T1, trimester 1; T2, trimester 2; T3, trimester 3; UTI, urinary tract infection; Vs., versus; WGA, weeks gestational age; WPPSI, wechsler preschool and primary scale of intelligence; WRAVMA, wide range assessment of visual motor abilities.

SupplementaryTable S2. Characteristics of Included Studies on the Long-Term Safety of Neonatal Paracetamol Exposure

Study	Design	Aim	Population	Sample Size	Exposure Assessment	GA (weeks)	BW (kg) Mean (SD)	Comorbidities	Underlying Condition	PNA at Exposure (days)	Dose (mg/kg), Dose Interval	Duration (days)	Cumulative Dose (mg/kg)	Outcome Assessed	Age at Outcome Assessment (years)	Method of Assessment	Results	Conclusions
Bauer, 2013 [22]	Ecologic	If use of paracetamol in early childhood might be a risk factor for ASD.	Cases 9 countries with data on circumcision and ASD after 1995. Controls 12 countries with data on circumcision and ASD before 1995.	N/A	Circumsision data/rate	N/R	N/R	N/R	N/R	N/R	Paracetamol			ASD	N/R	N/R	Strong correlation (r = 0.98) between circumsision and ASD. Change of 10% in the population circumcision rate associated with an increase in ASD prevelence of 1.01/1,000 persons.	Country- and state-level correlations between indicators of prenatal and neonatal paracectamol exposure and ASD.
											N/R	N/R	N/R					
											Other Pharmacotherapy, Placebo or Standard Practice							
											N/R	N/R	N/R					
Oncel, 2017 [50]	RCT Follow-Up	The effects of paractamol vs. ibuprofen for closure of PDA on NDV outcomes.	Cases Preterm infants with PDA on oral paracetamol	30	Chart Review	28 (1.7)	0.99 (0.21)	RDS: 26 [86.7] Pneumothorax: 2 [6.7] Sepsis: 6 [20] NEC: 1 [3.3] IVH: 2 [6.7] ROP: 3 [10]	PDA	2-3	Paracetamol			NDV outcomes; hearing and vision status.	1.5-2	BSID, MDI, PDI<br><br>Audiologist, Opthomalogist	Paracetamol vs. Ibuprofen Mean MDI score; PDI score; NDV impairment rate: 81.7 (16.6) vs. 82.1 (14.3 (p = 0.92); 81.7 (15.6) vs. 81.9 (13.1) (p = 0.96); 30% vs. 32.3% (p = 0.84).<br><br>Regression Analysis No effects of the risk factors on CP or NDI (p > 0.05).	No evidence for significant difference in NDV outcomes for paracetamol vs. ibuprofen at 1.5 to 2 years.
											15, every 6 hours	3	N/R					
			Controls Preterm infants with PDA on oral ibuprofen	31		27.6 (1.9)	0.98 (0.18)	RDS: 30 [96.8] Pneumothorax: 1 [3.2] Sepsis: 8 [25.8] NEC: 0 IVH: 1 [3.2] ROP: 2 [6.5]			Other Pharmacotherapy, Placebo or Standard Practice							
											Ibuprofen: 10, 5, 5, every 24 hours.	2	N/R					
			Cases	23				N/R	PDA	1-4	Paracetamol				2			

Juujarvi, 2021 [51]	RCT Follow-Up	Long-term outcomes and safety of IV paracetamol for PDA closure in preterm infants.	Preterm infants with PDA on IV paracetamol.		Chart Review	23+5 to 31+6	1.22 (0.43)	20, 7.5, every 6 hours	4	126	NDV testing using the GDMS	Parental Questionnaire	No significant difference in GDMS score, 24.0 (2.4) vs. 23.5 (2.3) (p = 0.47). .	No long-term adverse reactions of IV paracetamol was detected two years later.
			Controls Preterm infants with PDA on IV placebo.	21		23+5 to 31+6	1.12 (0.34)	Other Pharmacotherapy, Placebo or Standard Practice						
								Placebo: 0.45% saline	4	N/R				

Mean (SD); Number [percent]

ASD, autism spectrum disorder; BSID, bayley scales of infant development; BW, birweight; CP, cerebral palsy; GA, gestational age; GDMS, griffiths mental development scales; IV, intravenous; IVH, intraventricular hemorrhage; Kg, kilograms; MDI, major depression inventory; Mg, milligrams; NA, not applicable; NDI, neurodevelopmental impairment; NDV, neurodevelopmental; NEC, necrotizing enterocolitis; N/R, not reported; PDA, patent ductus arteriosus; PDI, pain disability index; PNA, postnatal age; RCT, randomized controlled trial; RDS, respiratory distress syndrome; ROP, retinopathy of prematurity;

SD, standard deviation, Vs., versus.

Supplementary Table S3. Risk of Bias Assessment of the Included Observational Studies using the NOS [1] [15]

Study	NOS Criteria									
	Selection				Comparability		Outcome			Total Score
	Exposed Cohort Representative	Selection of Non-Exposed Cohort	Exposure Ascertainment	Outcome Not Present at Start of Study	Adjustment for Important Risk Factors	Adjustment for Other Risk Factors	Outcome Assessment	Follow-Up Length	Loss to Follow-Up	
Stergiakouli, 2016 [23]	1	1	1	1	1	0	1	1	0	7
Bertoldi, 2020 [24]	1	1	0	1	1	1	1	1	1	8
Ji, 2018 [25]	0	1	1	1	1	1	1	1	1	8
Ji, 2020 [26]	1	1	1	1	1	1	1	1	1	9
Golding, 2020 [27]	1	1	0	1	1	0	1	1	1	7
Vlenterie, 2016 [28]	1	1	0	1	1	1	0	1	1	7
Ystrom, 2017 [29]	1	1	0	1	1	1	1	1	1	8
Liew, 2016 [30]	1	1	1	1	1	1	1	1	1	9
Rifas Shiman, 2020 [31]	1	1	1	1	1	1	0	1	1	8
Streissguth, 1987 [32]	1	1	1	1	1	1	1	1	1	9
Thompson,	1	1	1	1	1	1	0	1	1	8

2014 [33]									
Petersen, 2018 [34]	1	1	0	1	1	1	1	1	0	7
Andersen, 2012 [35]	1	1	1	1	1	1	1	1	1	9
Garcia-Marcos, 2009 [36]	1	1	0	0	1	1	0	1	1	6
Piler, 2018 [37]	1	1	0	1	1	0	0	1	1	7
Shaheen, 2002 [38]	1	1	0	1	1	1	0	1	1	7
Jedrychowski, 2011 [39]	1	1	1	1	1	1	1	1	0	8
Persky, 2008 [40]	0	1	0	1	1	1	0	0	1	5
Rebordosa, 2008 [41]	1	1	1	1	1	1	1	1	1	9
Magnus, 2016 [42]	1	1	0	1	1	1	1	1	1	8
Sordillo, 2015 [43]	1	1	0	1	1	1	0	1	1	7
Bakkeheim, 2011 [44]	1	1	1	1	1	0	1	1	1	7

Ernst, 2019 [45]	1	1	1	1	1	1	0	1	0	7
Fisher, 2016 [46]	1	1	0	1	1	0	1	1	0	6
Lind, 2017 [47]	1	1	0	1	1	1	1	1	0	7
Snijder, 2012 [48]	1	1	0	1	1	1	1	1	1	8
Shaheen, 2010 [49]	1	1	1	1	1	1	1	1	1	9

[1] Newcastle-Ottawa Quality Assessment Scale for Cohort Studies

Supplementary Table S4. Risk of Bias Assessment of the Included Ecologic Study using the Modified Tool for Assessing the Quality of Modern Cross-Sectional Ecologic Studies [16-18]

**Study**	**Tool for Assessing the Quality of Moderm Cross-Sectional Ecologic Studies Criteria**										
	**Study Design and Focus**				**Statistical Methodology**			**Quality of Reporting**			**Total Score[1]**
	Sample Size	Level of Inference	Pre-Specification of Ecological Units	Validity of Statistical Inferences	Use of Covariates	Spatial Effects	Proper Adjustment for Covariates	Statement of Study Design	Justification of Study Design	Discussion of Cross-Level Bias and Limitations	
Bauer, 2013 [22]	4	4	4	1	0.5	0	0.5	1	1	1	17

[1] The maximum score is 21. A score of $\geq$ 15 suggests high study quality

Supplementary Table S5. Certainty of the Evidence on the Long-Term Safety of Prenatal Paracetamol Exposure [20]

GRADE[1] Domain	Judgement	Concerns of Certainty Domains
**Methodological Limitations**	All but three observational epidemiologic or cohort studies were high-quality (score ≥ 7) based on the eight items assessed (representativeness of the exposed cohort, selection of the non-exposed cohort, ascertainment of exposure, demonstration that the outcome of interest was not present at the start of study, comparability, ascertainment of the outcome, length of follow-up, and adequacy of follow-up cohort).<br>The one included ecologic study was also high-quality (score ≥ 15) based on the three items assessed (study design and focus, statistical methodology and quality of reporting).<br>Therefore, we judged the methodological limitations of the studies to be not serious.	Not serious
**Indirectness**	The patients and interventions/exposures in the studies all provided direct evidence to the clinical question. All exposures/interventions were to paracetamol. Outcomes, for example, neurodevelopmental adverse outcomes, were assessed using different measures in the included studies (the Strengths and Difficulties Questionnaire, Wide Range Assessment of Visual Motor Abilities, NTERGROWTH-21st Neurodevelopment Assessment, Behavior Rating Inventory of Executive Function, among others).<br>We judged the evidence to have no serious indirectness but noted variation in outcome measurement.	Not serious
**Imprecision**	The total number of participants included in the studies was 505, 610[2]<br>The 95% Confidence Intervals reported in many studies crossed the line of no difference.<br>Therefore, we judged the evidence to have serious imprecision.	Serious
**Inconsistency**	The direction and magnitude of effect differed among the included studies. For studies that reported an increased occurrence or risk of adverse long-term safety outcomes, most observed small to moderate increased occurrence or risk. We judged the evidence to have serious inconsistency.	Serious
**Publication Bias**	Publication bias was not strongly suspected as our search was comprehensive and both positive and negative studies were published.	Not serious

[1] Grading of Recommendations, Assessment, Development and Evaluations

[2] The study by Bauer et al. was excluded from this number as individual participant numbers were not provided (country-level data was used); Several studies reported varying numbers of participants for outcomes that were assessed at different ages i.e., current asthma at three and seven years. When this occurred, we used the lowest number to conservatively calculate the total number of included participants.

Supplementary Table S6. Summary of Findings on the Long-Term Safety of Prenatal Paracetamol Exposure [20]

Outcome	Effect	Number of Participants	Certainty in the Evidence
Long-term safety (neurodevelopmental adverse events, atopic disorders, and reproductive disorders)	The direction of effect was unclear. For studies that reported increased occurrence or risk of adverse long-term safety outcomes, most observed small to moderate increased occurrence or risk.	505, 610[1] (27 observational epidemiologic or cohort studies, one ecologic study)	Low ⊕OOO (due to the included studies being observational, serious imprecision and serious inconsistency)

[1] The study by Bauer et al. was excluded from this number as individual participant numbers were not provided (country-level data was used); Several studies reported varying numbers of participants for outcomes that were assessed at different ages i.e., current asthma at three and seven years. When this occurred, we used the lowest number to conservatively calculate the total number of included participants.

Supplementary Table S7. Certainty of the Evidence on the Long-Term Safety of Neonatal Paracetamol Exposure [20]

GRADE[1] Domain	S	Concerns of Certainty Domains
**Methodological Limitations**	Of the two included randomized controlled trials (RCTs), one had a low risk of bias and the other had some concerns, based on the five items assessed (risk of bias due to: randomization, deviations from the intended interventions, missing outcome data, measurement of the outcome, and selection of the report result).<br>The one included ecologic study was also high-quality (score ≥ 15) based on the three items assessed (study design and focus, statistical methodology and quality of reporting).<br>Therefore, we judged the methodological limitations of the studies to be not serious.	Not serious
**Indirectness**	The patients and interventions/exposures in the studies all provided direct evidence to the clinical question. All exposures/interventions were to paracetamol. Outcomes, for example, neurodevelopmental adverse outcomes, were assessed using different measures in the included studies (Bayley Scales of Infant Development and Griffiths Mental Development Scales, among others).<br>We judged the evidence to have no serious indirectness but noted variation in outcome measurement.	Not serious
**Imprecision**	The total number of participants included in the studies was 105[2]<br>Only two of the three studies reported a 95% Confidence Interval (CI). In one of the two studies, the 95% CIs crossed the line of no difference.<br>Therefore, we judged the evidence to have very serious imprecision.	Very serious
**Inconsistency**	The direction of effect differed among the three included studies. The two RCTs showed no effect, but the ecologic study found a strong correlation between neonatal paracetamol exposure and autism spectrum disorder.<br>We judged the evidence to have serious inconsistency.	Serious
**Publication Bias**	Publication bias was not strongly suspected as our search was comprehensive and both positive and negative studies were published.	Not serious

[1] Grading of Recommendations, Assessment, Development and Evaluations

[2] The study by Bauer et al. was excluded from this number as individual participant numbers were not provided (country-level data was used)

Supplementary Table S8. Summary of Findings on the Long-Term Safety of Neonatal Paracetamol Exposure [20]

Outcome	Effect	Number of Participants	Certainty in the Evidence
Long-term safety (neurodevelopmental adverse events, atopic disorders, and reproductive disorders)	The direction of effect was toward no effect. The two randomized controlled trials (RCTs) showed no effect, but the ecologic study found a strong correlation between neonatal paracetamol exposure and autism spectrum disorder. Due to the methodological limitations associated with the ecologic study design, we are severely limited in our ability to make causal inferences based on the study results.	105[1] (two RCTs, one ecologic study)	Moderate ⊕⊕ OO (due to very serious imprecision and serious inconsistency)

[1] The study by Bauer et al. was excluded from this number as individual participant numbers were not provided (country-level data was used)

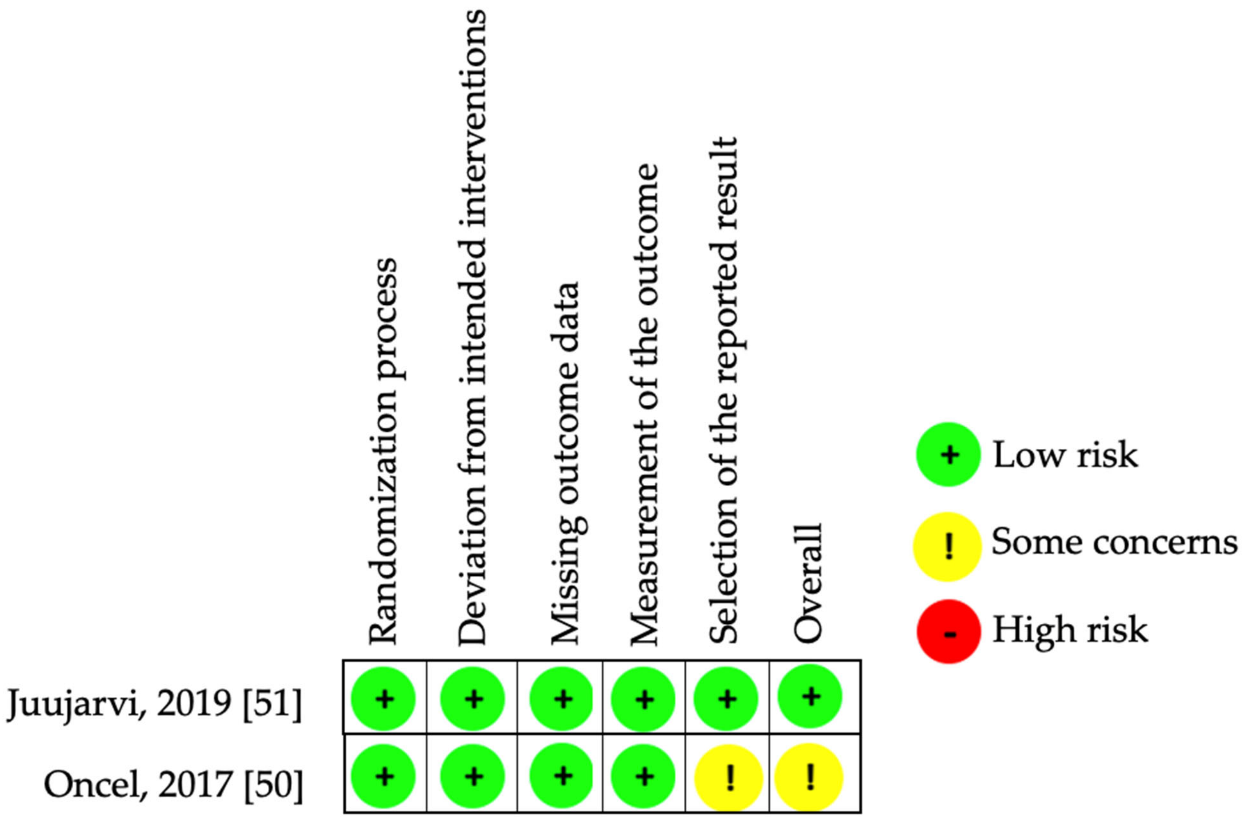

Supplementary Figure S1. Risk of Bias Assessment Summary – Review Authors' Judgements About Each Risk of Bias Item in RoB 2 [19] for the Included Randomized Controlled Trials
